# The macrophage at the intersection of immunity and metabolism in obesity

**DOI:** 10.1186/1758-5996-3-29

**Published:** 2011-10-28

**Authors:** M Constantine Samaan

**Affiliations:** 1 Division of Pediatric Endocrinology, Department of Pediatrics, McMaster Children's Hospital, McMaster University, 1200 Main Street West, Hamilton, Ontario, Canada

**Keywords:** Obesity, type 2 diabetes, inflammation, macrophage, cytokines, chemokines, muscle, adipose tissue, liver

## Abstract

Obesity is a worldwide pandemic representing one of the major challenges that societies face around the globe. Identifying the mechanisms involved in its development and propagation will help the development of preventative and therapeutic strategies that may help control its rising rates.

Obesity is associated with chronic low-grade inflammation, and this is believed to be one of the major contributors to the development of insulin resistance, which is an early event in obesity and leads to type 2 diabetes when the pancreas fails to keep up with increased demand for insulin. In this review, we discuss the role of macrophages in mediation of inflammation in obesity in metabolic organs including adipose tissue, skeletal muscle and liver. The presence of immune cells at the interface with metabolic organs modulates both metabolic function and inflammatory responses in these organs, and may provide a potential therapeutic target to modulate metabolic function in obesity.

## Introduction

More than one billion people around the world are overweight or obese, and one in eight of those is a child[1]. Another worldwide pandemic is type 2 diabetes, with around 350 million people affected[2] and this is mostly related to obesity. The burdens of obesity and diabetes with their co-morbidities on the individual, family, community, health care systems, and society at large is one of the biggest challenges that societies face around the globe[3]. Understanding the mechanisms involved in the development of the two conditions paves the way for design of preventative and therapeutic strategies that may stem their progress.

In this review, we highlight the role of the macrophage, an immune cell, in the development of obesity-mediated inflammation. We review the evidence for the intersection of nutrient and cytokine sensing in immune and metabolic organs, and assess the evidence for presence and actions of macrophages in metabolic organs.

### Nutrient sensing, cytokine signaling and inflammation in obesity

It is well established that obesity is associated with inflammation[4-15], but this inflammation is different from that seen in infection or autoimmunity. First, obesity-associated inflammation does not fulfill the usual description of acute inflammation with redness, heat, swelling, and pain but follows a more chronic and low-grade course[16-19]. Second, inflammation in obesity is a systemic process that affects many organs, but may begin in one or more organs. Inflammation starts in adipose tissue as it expands with excess fat and caloric intake, and involves activation of inflammatory pathways in cells by nutrient-sensing and cytokine signaling. Nutrient sensing occur via pattern recognition receptors including membrane-based innate immune receptors known as toll-like receptors 2 and 4 (TLR2 & TLR4), and intracellular pathogen sensing NOD-like receptors [20-24]. These receptors were originally thought to be involved only in pathogen sensing, but recently were found to sense fatty acids.

**Figure 1:**
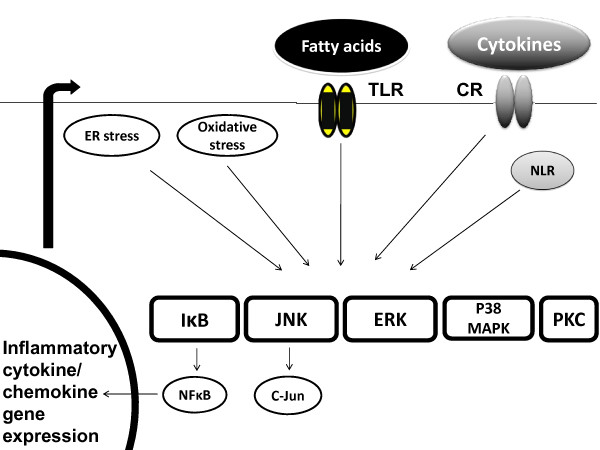
**Fatty acids and cytokines utilize similar downstream signaling pathways to activate inflammatory response in obesity**. Saturated fatty acids signal via toll-like receptors (TLRs), and cytokines signal via respective cytokine receptors (CR) to activate MAPK enzymes including JNK, ERK, and p38MAPK along with IκB and protein kinase C (PKC) pathways. Other mechanisms include activation of these pathways by NOD-like receptors (NLRs), production of reactive oxygen species and oxidative stress, and endoplasmic reticulum stress (ER stress). The activation of these pathways leads to production of transcription factors including NFκB and C-Jun that enter nucleus and bind specific sequences on gene promoters, and lead to transcription of inflammatory cytokine and chemokine genes. The cytokines produced will act in an autocrine, paracrine and endocrine manner and interfere with insulin signaling. They will also stimulate further cytokine production that will propagate the activation of these pathways, leading to further inflammation.

In addition, cytokines are produced in response to inflammatory stimuli from different organs, and can act in an autocrine, paracrine or endocrine fashion[25-30]. Fatty acids and cytokines converge to activate the same downstream inflammatory pathways. The activation of these pathways leads to production of transcription factors that enter the nucleus and activate inflammatory cytokine gene expression and cytokine production, leading to propagation of inflammation[31-34](Figure 1).

Recently, an intriguing mechanism was proposed as an explanation for obesity. CNS deletion of Toll-like receptor 4(TLR4) adaptor MyD88 protected mice from high fat diet-mediated obesity. Intracerebroventricular administration of saturated fatty acid palmitate did not elicit leptin or insulin resistance and did not increase hypothalamic inflammatory cytokines. This indicates that high fat diet in part acts centrally even before meaningful weight gain to trigger leptin and insulin resistance. This leads to dysregulation of anorexigenic effects of leptin and insulin, and initiation and progression of obesity[35]. Hypothalamic inflammation as a starting point for obesity is an interesting mechanism that requires further study [36,37].

### Immune cells in metabolic organs drive inflammation in obesity

While many mechanisms leading to inflammation in obesity are not completely understood, it is clear that the interaction between immune and metabolic cells initiates and propagates the inflammatory response.

Adipose tissue expansion in obesity outpaces its blood supply, resulting in adipose tissue hypoxia and activation of inflammatory responses, with production of factors including Hypoxia Inducible Factor-1α [HIF-1α][38]. It is also associated with altered adipokine production with enhanced production of leptin and resistin, and reduced production of adiponectin [39-50].

In addition, inflammation results in secretion of cytokines and chemokines. This will activate adipose tissue T- lymphocytes and resident macrophages, which are present in adipose tissue under physiological conditions. This then leads to secretion of pro-inflammatory cytokines and chemokines from these cells that attract immune cells including other T-lymphocytes, neutrophils, and monocytes[16,18,51-60]. Once in adipose tissue, monocytes differentiate to macrophages, and start secreting cytokines that propagate local inflammation in adipose tissue.

## Inflammatory pathway activation drives inflammation and insulin resistance in obesity

Inflammation mediated by increased cytokine production and excess fatty acids or 'lipotoxicity' activate inflammatory pathways in immune and metabolic cells. These pathways include Mitogen Activated Protein Kinase (MAPK) pathway members including c-jun amino terminal kinase (JNK), Extracellular Regulated Kinase (ERK), and p38MAPK. In addition IκB kinase [61-69] and protein kinase C (PKC)[70-73] pathways are also activated. The activation of these pathways leads to interference with insulin signaling leading to insulin resistance, an early event in obesity[16,53-57]. Insulin resistance leads to mitigation of anti-lipolytic effect of insulin on adipose tissue, and lipolysis ensues with local fatty acid release triggering further local inflammation. What begins as a local process eventually exceed the capacity of adipose tissue to contain it, and cytokines and free fatty acids are released into the circulation and reach metabolic organs including skeletal muscle and liver[74,75].

**Figure 2:**
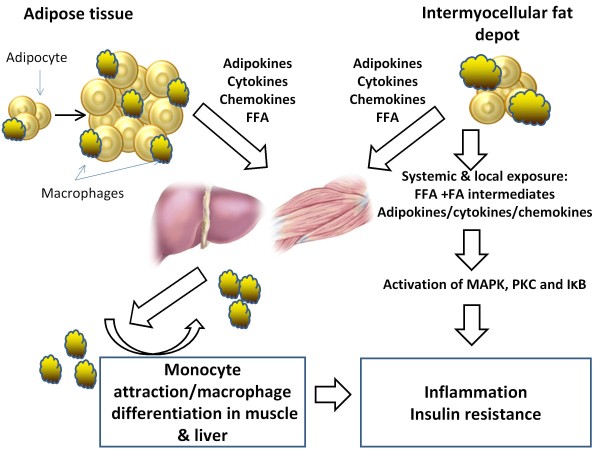
**The model for macrophage-metabolic interactions and inflammation in obesity**. Adipose tissue expansion in obesity results in an inflammatory state characterized by production of adipokines, cytokines, and chemokines. This is also associated with free fatty acid [FFA] release via increased lipolysis. Activation of resident or 'M2' adipose tissue macrophages leads to secretion of inflammatory cytokines and chemokines; this will lead to attraction of monocytes that differentiate to macrophages. Fatty acids and cytokines spill into circulation, and get to metabolic tissues including skeletal muscle and liver. In addition, the expansion of the intermyocellular fat depot also attracts macrophages, and this is associated with secretion of pro-inflammatory cytokines, chemokines and FFA release that occur locally. The exposure of muscle to cytokines, chemokines and fatty acids from systemic and local sources triggers an inflammatory response characterized by activation of inflammatory pathways, upregulation of gene expression, and synthesis and secretion of inflammatory cytokines and chemokines. The attracted macrophages will in turn secrete inflammatory molecules, which signal through inflammatory pathways propagating this inflammatory response and resulting in muscle inflammation and insulin resistance. JNK = c-jun amino terminal kinase; PKC = protein kinase C.

In skeletal muscle, this is compounded by the presence of Intermyocellular Fat Depot [IMFD] that expands with obesity and harbors immune cells including macrophages[76](Figure 2).

In addition, fatty acid uptake into muscle cells leads to accumulation of intracellular triglycerides, which act as a sink to protect muscle from lipotoxicity. Interestingly, athletes have excess triglyceride stores in skeletal muscle, but demonstrate enhanced insulin sensitivity. This 'athlete's paradox' hints to the fact that triglycerides are not the culprits in mediating inflammation and insulin resistance, but it is metabolic intermediates like ceramide and diacylglycerol generated intracellularly that interfere with insulin signaling and trigger inflammation[77-79].

The deposition of fat into the liver leads to fatty liver with activation of inflammatory pathways, secretion of inflammatory cytokines, monocyte attraction and macrophage generation, which in turn leads to hepatic inflammation and insulin resistance [32,80].

The initial response to compensate for insulin resistance is via increased insulin production by pancreatic β-islet cells, leading to hyperinsulinemia. Eventually, the pancreas fails to maintain insulin production in the face of steadily increasing insulin demand, leading to the development of type 2 diabetes.

### Inflammation is associated with macrophage infiltration of metabolic organs

The detection of enhanced expression of Tumor Necrosis Factor-α [TNF- α], a prototypical inflammatory cytokine, in adipose tissue in obese mice provided first clues to presence of inflammation in obesity[81]. This was then confirmed in obese and diabetic humans[4]. The source of TNF-α was initially presumed to be the adipocyte, but it was eventually found to be from an immune cell, the macrophage[16,18,82-85]. These cells produce multiple cytokines and chemokines in obesity that modulate function of metabolic organs leading to inflammation and insulin resistance[83-85].

Macrophages are mononuclear phagocytic cells that are part of the innate immune system, an evolutionarily conserved defense system with cells placed at ports of entry of pathogens and other environmental threats to the body[86-88]. One function for these cells is sampling antigens as they enter the body and then either destroy them by an 'innate response' with no memory kept of the encounter, or present the antigen to the T-lymphocytes to mount an adaptive immune response[27,86]. The precursors of macrophages, the monocytes, are generated in the bone marrow. These cells are recruited to adipose tissue by signals from adipocytes and adipose tissue macrophages[89,90]. Once in the adipose tissue, the monocytes differentiate to macrophages[91].

## Phenotypic characterization of macrophages in metabolic organs

### Adipose tissue

Early in obesity, adipocytes are predominantly responsible for producing chemokines and macrophages are involved in producing cytokines, but both cells are capable of producing these molecules, and can modulate function of metabolic organs leading to inflammation and insulin resistance[83-85]. Adipose tissue macrophages are present in two main subtypes. The resident or 'M2' macrophages are present in almost all organs in the body as resident cells under physiological conditions, where they serve to maintain tissue homeostasis[92-95]. Under normal physiological states, 5-10% of adipose tissue cells are resident M2 macrophages. These cells are distinguished by being responsive to IL-4 and IL-13 and their ability to secrete anti-inflammatory cytokines like IL-10.

In addition, M2 macrophages upregulate production of Arginase I enzyme, reducing nitric oxide synthesis and inflammation via metabolizing arginine to ornithine [92,93,96-100]. Arginase I gene expression is stimulated by IL-4 and Signal Transducer and Activator of Transcription-6 (STAT-6) axis[101]. This in turn signals via the master regulator of adipogenesis and fatty acid sensor Peroxisome Proliferator-Activated Receptor gamma (PPARγ).

PPAR/RXR heterodimers bind to specific sequences at the Arginase I gene enhancer region activating its expression[102]. PPARγ is essential for attainment and preservation of M2 macrophage phenotype, as its deletion in macrophages leads to excess adiposity in mice on high fat diet. It also results in insulin resistance in skeletal muscle and liver in these mice. When adipocytes are co-incubated with PPARγ knockout macrophages, they become insulin resistant, which argues for factors produced by these macrophages, including IL-4 among others, that modulate metabolic function in adipose tissue, liver and skeletal muscle[102,103].

On the other hand, bone marrow-derived monocytes migrating into obese adipose tissue and exposed to fatty acids and cytokines differentiate to an inflammatory or 'M1' macrophages [89,91,104,105]. M1 macrophages are responsive to interferon gamma and lipopolysaccharide, and are capable of producing pro-inflammatory cytokines and nitric oxide. Their numbers increase several folds with obesity and high fat feeding[18].

The M1/M2 divide is a simplistic view of the reality of macrophage existence in different organs[106,107]. Macrophages very likely exist in multiple intermediate phenotypes depending on local tissue microenvironment[98,99], and are able to respond to local cues and shift their phenotype to maintain local tissue homeostasis.

Another consideration is that while M1 macrophages in obese adipose tissue originate mainly from bone marrow [90], it is possible that other cells may contribute to this pool. The pre-adipocyte may be a potential source of macrophages in obese adipose tissue, as these cells share common capabilities with macrophages in response to obesogenic environments.

Pre-adipocytes reside in stromal vascular fraction within adipose tissue, and are bathed in the same cocktail of nutrients and cytokines that influence their interactions with other cells in that environment[108-110]. Macrophages are capable of storing lipids as seen in foam cells present in atherosclerotic plaques, and foam-like cells are also seen in obese adipose tissue[111-113]. Pre-adipocytes injected into peritoneal cavity of mice can act like macrophages, with phagocytosis of microorganisms and posses antimicrobial actions via generation of reactive oxygen species [108,110]. These abilities disappear when cells differentiate to mature adipocytes.

Pre-adipocytes can also differentiate to macrophages with expression of many macrophage markers[108], and this is probably due to direct physical contact between pre-adipocyte and macrophage. In addition, the transcriptional profile of pre-adipocyte is in fact closer to the macrophage than to the adipocyte, and these cells produce many common products to both including cytokines, chemokines, and adhesion molecules [108,110,114,115].

More recently, weight loss in mice previously fed high fat diet lead to infiltration of adipose tissue with M2 macrophages. This was mediated by lipolysis, and macrophages were acting as neutralizers of effects of excess fat in obese adipose tissue [74]. This is in contrast to when weight gain leads to the infiltration of M1 macrophages, and argues for a robust immune response to local adipose tissue microenvironment, and this M2 infiltration may be an attempt to protect adipose tissue from mounting an inflammatory response with excess local fatty acid concentrations. The ultimate aim of inflammation is the induction of tissue remodeling and to restore homeostasis, and M2 macrophages act to initiate and propagate this process with weight loss[116,117], and depending on local tissue microenvironment, there is potential plasticity in cell phenotype.

## Skeletal muscle

In skeletal muscle, circulating cytokines and fatty acids and those produced locally from IMFD [e.g. TNFα, IL-6] jointly affect muscle. Macrophages infiltrate IMFD when it expands in obesity [18], and as the IMFD depot is in immediate vicinity of skeletal muscle, it is likely that muscle-macrophage crosstalk will impact both cells. Insulin resistance in muscle is caused by several mechanisms including fatty acid oxidation defects due to effects on mitochondrial biogenesis, oxidative stress, accumulation of lipid intermediates in muscle, and effects of pro-inflammatory cytokine on insulin signaling [57,61,118-122].

Macrophage products from saturated fatty acid treatment *in-vitro *causes skeletal muscle insulin resistance[123]. The question whether macrophages infiltrate skeletal muscle in obesity is important, as muscle takes up to 75% of carbohydrate intake after a meal, making it a major determinant of postprandial glycemic status.

There is increasing evidence that macrophages may infiltrate skeletal muscle in obesity. In rodents, high fat feeding increases macrophage infiltration in muscle compared to normal chow fed animals [124]. In addition, deletion of PPARγ in myeloid cells, a master regulator of adipogenesis and inflammation, also resulted in increased macrophage and dendritic cell markers in muscle [125]. Macrophage markers have been documented mainly in IMFD, and rarely between myofibrils with high fat feeding using immunohistochemistry [18,124] and bone marrow transplant experiments [126]. In the latter case, macrophages were detected at the muscle-fat junction, raising the question of whether their location denotes a non-directional chemokinetic response, or a true directional, chemotactic response to factors produced by muscle.

Furthermore, dendritic cell were detected in muscle from high fat fed mice, and fatty acid treatment of bone marrow derived macrophages [BMDM] and dendritic cells [BMDC] induced inflammation in the BMDC and not BMDM [126].

In human studies, macrophages were detected in skeletal muscle from obese non-diabetic subjects and this was positively associated with body mass index and negatively associated with insulin sensitivity [127]. Macrophages were also detected in human muscle from subjects with normal glucose tolerance but at much lower levels than in adipose tissue [128].

However, other studies failed to demonstrate the presence of macrophage markers using microarrays of muscle from high fat fed mice [16] and no increase of macrophage markers in muscle was noted in severely obese humans undergoing lifestyle intervention program [129].

While the above studies provide evidence for and against the infiltration of skeletal muscle by macrophages, there are also challenges to pinpoint the mechanisms of muscle-macrophage interaction in obesity. First, whole tissue analysis for presence of macrophage markers does not necessarily clarify the location of these macrophages, as they could be adherent to the endothelium within blood vessel lumen rather than infiltrating into muscle tissue when tissues are prepared for analysis. Second, a significant challenge is to clarify if these macrophages are in fact infiltrating within muscle fibers or are they mainly in the IMFD that expands in obesity and attracts macrophages. The potential mechanisms and pathways involved in macrophage recruitment to muscle remain incompletely understood.

## Liver

The accumulation of fat in the liver causes Non-alcoholic fatty liver disease (NAFLD) which is an important complication of obesity[130]. Resident liver macrophages [Kupffer cells] play a significant protective role in obesity by producing anti-inflammatory cytokines e.g. IL-10, and their depletion results in hepatic inflammation and insulin resistance[131]. In addition, obesity leads to macrophage recruitment to the liver via their Chemokine C-C receptor-2 (CCR2) in response to Chemokine C-C ligand-2 (CCL2) produced by hepatocytes, and these cells regulate hepatic lipid accumulation in the liver[132,133].

In addition, the depletion of resident liver macrophages [Kupffer cells] leads to protection from effects of high fat feeding-induced inflammation.

The association of liver inflammation via the production of inflammatory cytokines including TNFα and IL-6 has recently been also linked to the development of hepatocellular carcinoma via stimulation of STAT-3[134]. As obesity is associated with risk of several cancers, this is a novel area of research that requires further investigation to clarify the link between obesity, inflammation and cancer.

## Conclusions

In summary, obesity is associated with chronic low-grade inflammation, and macrophage crosstalk with metabolic organs mediates this inflammatory response. Macrophage precursors are recruited to metabolic organs in obesity, and produce several factors that lead to inflammation and insulin resistance in these organs.

The Inflammatory process seen in obesity is caused by the activation of several pathways activated by nutrients including fatty acids and cytokines. The collusion of these stimuli leads to interference with insulin signaling and insulin resistance (Figure 2), which is an early step on the path to type 2 diabetes.

Defining the mechanisms by which different pathways and molecules modulate progression of inflammation in obesity holds the promise of developing interventions that may help hundreds of millions of people around the world struggling with obesity and type 2 diabetes.

## Competing interests

The author declares that they have no competing interests.
